# Immunohistochemical Demonstration of Tuft Cells in Human Acinar-to-Ductal Metaplasia and Pancreatic Intraepithelial Neoplasia

**DOI:** 10.3390/biomedicines13081944

**Published:** 2025-08-08

**Authors:** Kensuke Nakanishi, Mitsuaki Ishida, Kohei Taniguchi, Kenta Hosomi, Jun Arima, Atsushi Tomioka, Mitsuhiro Asakuma, Yoshiharu Miyamoto, Ko Fujimori, Yoshinobu Hirose, Sang-Woong Lee

**Affiliations:** 1 Department of General and Gastroenterological Surgery, Osaka Medical and Pharmaceutical University, Osaka 569-8686, Japan; kensuke.nakanishi@ompu.ac.jp (K.N.); jun.arima@ompu.ac.jp (J.A.); atsushi.tomioka@ompu.ac.jp (A.T.); mitsuhiro.asakuma@ompu.ac.jp (M.A.); sang-woong.lee@ompu.ac.jp (S.-W.L.); 2 Department of Pathology, Osaka Medical and Pharmaceutical University, Osaka 569-8686, Japan; yoshinobu.hirose@ompu.ac.jp; 3 Centre for Medical Research & Development, Division of Translational Research, Osaka Medical and Pharmaceutical University, Osaka 569-8686, Japan; kohei.taniguchi@ompu.ac.jp; 4 Department of Pathobiochemistry, Osaka Medical and Pharmaceutical University, Osaka 569-1094, Japan; ompu72123058@s.ompu.ac.jp (K.H.); ko.fujimori@ompu.ac.jp (K.F.); 5 Department of Gastroenterological Surgery, Ishinkai Yao General Hospital, Osaka 581-0036, Japan; yoshi9637@gmail.com

**Keywords:** pancreas, tuft cells, acinar-to-ductal metaplasia, pancreatic intraepithelial neoplasia

## Abstract

**Background/Objectives**: Acinar-to-ductal metaplasia (ADM) refers to the dedifferentiation or transdifferentiation of pancreatic acinar cells. Recently, ADM has received considerable attention as a potential precursor of pancreatic tumours. Previous studies in mouse models identified tuft cells, chemosensory epithelial cells, in ADM and pancreatic intraepithelial neoplasia (PanIN), both considered precursor lesions of pancreatic ductal adenocarcinoma (PDAC), but not in PDAC. We examined the presence of tuft cells in human ADM and PanIN. **Methods**: We analysed tissue samples from 29 patients (16 women, 13 men; median age 74 years) who underwent surgical resection for pancreatic tumours. Immunohistochemical staining for the tuft cell marker, POU2F3, was used to detect tuft cells in ADM and PanIN lesions. **Results**: ADM was present in all patients. POU2F3-positive tuft cells were observed in 46.4% of ADM lesions (327/705) but not in normal pancreatic acini. The number of POU2F3-positive tuft cells per PanIN area were significantly higher in low-grade PanIN (median, 2 cells; range, 0–20 positive cells) than in high-grade PanIN (median, 0 cell; range 0–4 positive cells) (*p* = 0.0050). The percentage of POU2F3-positive tuft cells per total cells in low-grade PanIN lesions (median, 1.1%; range 0–2.5%) was also significantly higher than that in high-grade PanIN lesions (median, 0%; range 0–1.1%) (*p* = 0.0044). **Conclusions**: Our results suggest that tuft cells emerge in human pancreatic acini during ADM, possibly as part of tissue repair following injury.

## 1. Introduction

Pancreatic ductal adenocarcinoma (PDAC) is one of the most fatal carcinomas worldwide. Pancreatic intraepithelial neoplasia (PanIN) is a potential precursor lesion to PDAC, and the genetic progression from PanIN to PDAC has been extensively analysed to understand the molecular mechanisms underlying PDAC development [1,2].

Acinar-to-ductal metaplasia (ADM) is a phenomenon in which pancreatic acinar cells undergo dedifferentiation or transdifferentiation, acquiring an embryonic progenitor phenotype and ductal markers [3,4,5]. Previously referred to as a tubular complex, ADM is histopathologically characterised by the widening of the acinar lumina, lined by flattened cells lacking visible zymogen granules, which resemble pancreatic ductules [6,7]. Various factors, such as *Kras* hyperactivation and inflammation, can induce ADM [4]. ADM is commonly observed in human pancreatic tissues with acute and/or chronic inflammation (pancreatitis) [3,4,5]. Its presence has also been reported in the surrounding tissues of resected pancreatic tumours, such as obstructive pancreatitis [7]. ADM is considered a regenerative process in which acinar structures are reconstructed from injury, as acinar cells exhibit significant plasticity [4,5]. Moreover, in a cerulein-induced acute pancreatitis mouse model, ADM is caused by inflammatory mediators secreted by macrophages, and it is a reversible event that facilitates regeneration [8,9]. Notably, several studies using a transgenic mouse model (*Pdx1*-Cre; *Kras*^LSL-G12D^) have demonstrated that ADM can cause PanIN, an initiating event in the development of PDAC; however, the precise pathway from ADM to PDAC via PanIN remains unclear in the human pancreas [4]. Accordingly, ADM has gained considerable attention as a potential precursor to PDAC.

Tuft cells are chemosensory epithelial cells present on the normal luminal surfaces of the alimentary and respiratory tracts [10]. These cells act as luminal sensors because they express taste receptors [10]. POU domain class 2 transcription factor 3 (POU2F3), also known as Oct11 or Skn1, is the most useful marker for tuft cells because it is the master regulator of tuft cell identity [10,11]. Tuft cells play an important role in the response to parasitic infections and antibacterial defence [10]. Tuft cells are responsible for tissue repair and injury [10]. Recent studies have revealed that tuft cells play pivotal roles in pancreatitis [10,12,13,14,15]. Recent evidence has shown that acinar cells can transdifferentiate into tuft cells in ADM regions in response to tissue injury [12,13]. Moreover, DelGiorno et al. demonstrated that tuft cells inhibited the development and acceleration of PanIN to PDAC in a *Kras*-induced pancreatic tumorigenesis mouse model [16]. Tuft cells appear in ADM and gradually decrease from ADM to PanIN; no tuft cells are present in PDAC [14,16]. However, these data were derived from a mouse model of pancreatitis or pancreatic tumours, and no data on the role of tuft cells in human ADM and PanINs are available.

Therefore, we aimed to evaluate the presence or absence of tuft cells in human ADM in obstructive pancreatitis and PanIN lesions by using the immunohistochemical staining for a specific human tuft cell marker, POU2F3, in resected human pancreatic tissues. We also discuss the potential roles of tuft cells in human pancreatitis and pancreatic lesions.

## 2. Materials and Methods

### 2.1. Patient Selection

We selected consecutive patients with pancreatic tumours who underwent surgical resection at the Department of General and Gastroenterological Surgery of the Osaka Medical and Pharmaceutical University Hospital between January 2022 and December 2023. Patients who underwent neoadjuvant chemotherapy and/or radiation therapy were excluded because these therapies may influence the presence of tuft cells in the pancreas.

This retrospective, single-institution study was conducted in accordance with the tenets of the Declaration of Helsinki, and the study protocol was approved by the Institutional Review Board of Osaka Medical and Pharmaceutical University Hospital (Approval number 2023-198). All data were anonymised. Informed consent was obtained from patients using the opt-out methodology, because the study was a retrospective study and archived samples were used with no risk to the participants. We did not include children. The inclusion criteria and opportunity to opt-out were provided on the institutional website (https://www.ompu.ac.jp/u-deps/path/img/file23.pdf (accessed on 4 July 2025)).

### 2.2. Histopathological Analysis

Surgically resected specimens were fixed in 10% buffered neutral formalin, and the cut sections were stained with haematoxylin and eosin. Two researchers (K.N. and M.I.) independently evaluated the histopathological characteristics by analysing all the haematoxylin and eosin slides.

ADM is defined as the widening of the acinar lumina lined by flattened cells without obvious zymogen granules [6,7]. PanIN is histopathologically defined as cuboidal to columnar cells containing intracytoplasmic mucin and is graded as low- or high-grade. Low-grade PanINs are flat or papillary proliferative lesions with located or pseudostratified nuclei and mild-to-moderate cytological atypia. Contrarily, high-grade PanINs are papillary or micropapillary proliferations with marked loss of polarity, irregular nuclear stratification, and the budding of cell clusters into the lumen, severe cytological atypia, and mitosis [1].

### 2.3. Immunohistochemical Analyses

Immunohistochemical analysis was performed using an autostainer (Leica Bond-III; Leica Biosystems GmbH, Nußloch, Germany). Anti-POU2F3 rabbit monoclonal antibody (E5N2D; ×200; Cell Signaling Technology, Danvers, MA, USA) was used. Squamous cells of the skin were used as positive controls for POU2F3 expression. Negative controls without the primary antibody were also used. Nuclear staining was considered positive for POU2F3.

One or two representative slides of all surgically resected specimens containing ADM were used for immunostaining. Consecutive sections were used for haematoxylin and eosin stain and immunohistochemical staining for POU2F3 to identify ADM and PanIN lesions in the immunohistochemical slides. The numbers of POU2F3-positive cells were counted per PanIN area (both low- and high-grades) in all the immunohistochemical slides. The percentage of POU2F3-positive cells was evaluated in 10 typical low- and high-grade PanIN lesions, each.

Two researchers (K.N. and M.I.) independently evaluated the immunohistochemical features. When there were discrepancies, a final decision was established by reassessment using a multi-headed microscope.

### 2.4. Statistical Analyses

The two correlations between the two groups were analysed using the Mann–Whitney *U* test. Statistical significance was set at *p* < 0.05.

## 3. Results

### 3.1. Patient Characteristics

Table 1 summarizes the clinicopathological features of the present cohort. This study included 16 women (55.2%) and 13 men (44.8%). The median age at the time of surgery was 74 years (range: 51–84 years). The tumour locations were as follows: pancreatic head in 11 patients (37.9%) and body and tail in 18 patients (62.1%). The histopathological diagnosis of pancreatic tumours was as follows: PDAC in seven patients (24.1%) (five women and two men; head and body and tail in three and four patients, respectively), intraductal papillary mucinous neoplasm (IPMN) with low-grade dysplasia in 10 patients (34.5%) (six women and four men; head and body and tail in three and seven patients, respectively), IPMN with high-grade dysplasia or associated invasive carcinoma in 10 patients (34.5%) (four women and six men; head and body and tail in five patients each), solid pseudopapillary neoplasm (one man; body and tail), and neuroendocrine tumour accompanying IPMN with low-grade dysplasia (one woman; body and tail) in one patient (3.4%).

### 3.2. Histopathological Characteristics

**Figure 1:**
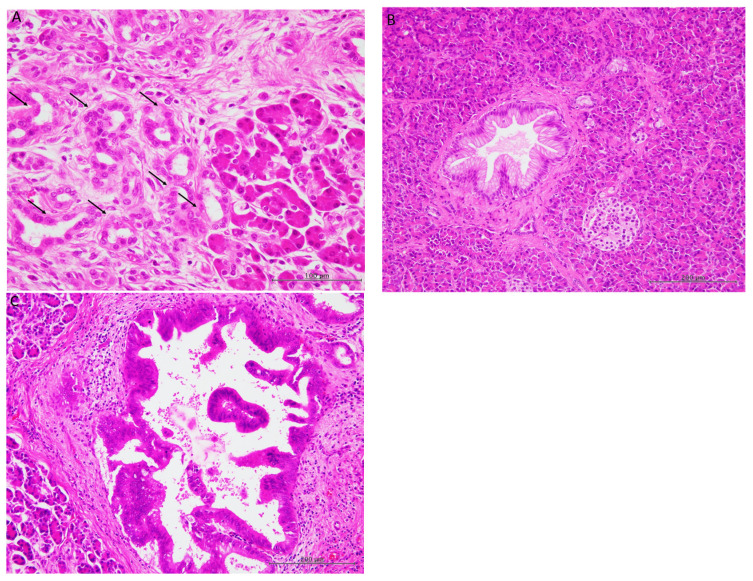
Histopathological features of the pancreas. (**A**) Acinar-to-ductal metaplasia in obstructive pancreatitis (arrows). The widening of the acinar lumina lined by flattered cells lacking visible zymogen granules, which resemble pancreatic ductules, are characteristic features of acinar-to-ductal metaplasia. Acinar cells containing zymogen granules are present adjacent to acinar-to-ductal metaplasia lesion (right side) (haematoxylin and eosin staining, ×400). (**B**) Low-grade pancreatic intraepithelial neoplasia (haematoxylin and eosin staining; original magnification, ×100). (**C**) High-grade pancreatic intraepithelial neoplasia (haematoxylin and eosin staining, ×100).

ADM was observed in obstructive pancreatitis lesions in the surrounding pancreatic tissues of all patients (Figure 1A).

Low-grade PanIN was noted in 28 patients (250 lesions) (except for one patient with IPMN-associated invasive carcinoma) (Figure 1B), and high-grade PanIN was observed in four patients with PDAC (14 lesions) (Figure 1C).

### 3.3. Immunohistochemical Characteristics

**Figure 2:**
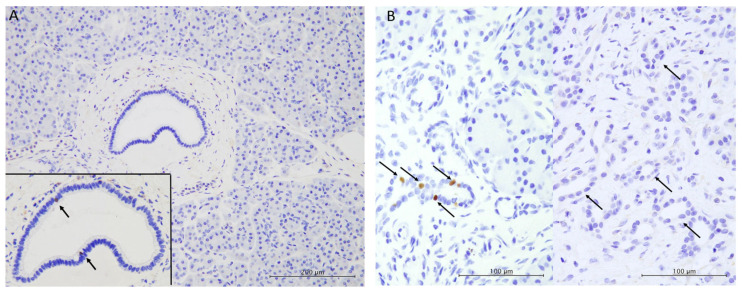

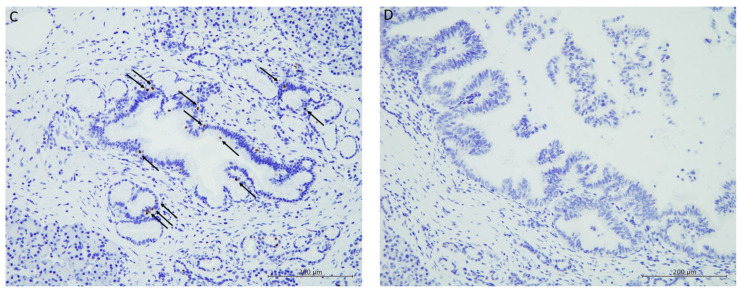
Immunohistochemical features of tuft cells in the human pancreas. (**A**) In non-neoplastic pancreatic tissue, a few POU2F3-positive tuft cells are present in the interlobular pancreatic duct (inset, arrows), but not in the acini (original magnification, ×100; inset, ×200). (**B**) Left: POU2F3-positive tuft cells are observed in acinar-to-ductal metaplasia (arrows). Right: No POU2F3-positive tuft cells are present in acinar-to-ductal metaplasia (arrows) (original magnification, ×400). (**C**,**D**) POU2F3-positive tuft cells are noted in low-grade pancreatic intraepithelial neoplasia (**C**, arrows) but not in high-grade intraepithelial pancreatic neoplasia (**D**) (original magnification, ×200).

In non-neoplastic pancreatic tissues, a few POU2F3-positive cells were present in the pancreatic ducts (from the interlobular ducts to the main pancreatic ducts). However, they were never observed in the acini, intercalated ducts, or excretory ducts (Figure 2A).

POU2F3-positive tuft cells were present in 46.4% of ADM lesions (327 of 705 lesions) (Figure 2B).

The number of POU2F3-positive tuft cells per PanIN area was significantly higher in low-grade PanIN (median, 2 POU2F3-positive tuft cells per lesion; range, 0–20 positive cells) than in high-grade PanIN (median, 0 per lesion; range, 0–4 positive cells) (*p* = 0.0050) (Table 2) (Figure 2C,D).

The percentage of POU2F3-positive tuft cells per total cells in low-grade PanIN lesions (median, 1.1%; range, 0–2.5%) was significantly higher than that in high-grade PanIN lesions (median, 0%; range, 0–1.1%) (*p* = 0.0044) (Table 2) (Figure 2C,D).

## 4. Discussion

This study demonstrated that POU2F3-positive tuft cells were present in 46.4% of ADM lesions, but not in the normal acini, and that the presence of tuft cells was significantly higher in low-grade PanIN than in high-grade PanIN in human pancreatic tissues for the first time. Recent studies using a mouse model of pancreatitis or pancreatic tumours demonstrated that tuft cells were present in ADM and PanIN but absent in PDAC and that acinar cells could transdifferentiate into tuft cells in the ADM in response to tissue injury [12,13,16]. Although direct evidence of transdifferentiation from acinar to tuft cells was not observed, our study showed for the first time that tuft cells appear in 46.4% of ADM lesions in human obstructive pancreatitis.

Tuft cells are chemosensory epithelial cells that express taste receptors and are present on the luminal surface of the alimentary and respiratory tracts [10]. In a normal pancreas, POU2F3-expressing tuft cells are present in the pancreatic ducts (from the interlobular ducts to the main pancreatic ducts) but not in the intercalated and excretory ducts and acini, according to the results of our study. This finding is consistent with that of a previous study on the distribution of tuft cells in the human pancreas using choline acetyltransferase [17]. Tuft cells have various functions, including antiparasitic and antibacterial responses, and secrete various physiologically active substances, such as interleukin-25 and acetylcholine [10]. Tuft cells play an important role in tissue repair and response to injury [10,13,16]. Previous studies using cerulein-induced pancreatitis mouse or *Kras*^G12D^ transgenic mouse models demonstrated that tuft cells could be transiently transdifferentiated from mature acinar cells, representing the plasticity of pancreatic acinar cells [13,16]. In the human pancreas, a previous report showed that immunostaining for phospho-epidermal growth factor receptors, a tuft cell marker, revealed the presence of tuft cells in human pancreatitis tissues [16]. Moreover, in situ RNA sequence analysis revealed the presence of tuft cells in human chronic pancreatitis tissues [18]. However, no detailed information on the location of tuft cells has been reported in previous studies [16,18]. According to the results of the present study, these tuft cells might be present in ADM. Moreover, a transgenic POU2F3-deficient mouse model showed that a lack of tuft cells leads to an increase in activated inflammatory cells via the secretion of prostaglandins by tuft cells [16]. Thus, tuft cells in ADM are thought to suppress inflammation, leading to tissue repair of pancreatic acini in mouse models [10,16] and human pancreatitis lesions. In addition, it is well known that chronic pancreatitis is a risk factor for PDAC [19]; thus, tuft cells may be associated with tumorigenesis in chronic pancreatitis.

PanIN is a possible precursor lesion of PDAC because it shares critical genetic abnormalities with PDAC, and histological progression from low- to high-grade PanIN parallels the accumulation of genetic abnormalities [20,21]. Low-grade PanIN is frequently observed in the human pancreas, especially in older individuals, whereas high-grade PanIN is typically associated with the presence of PDAC, and its presence without pancreatic tumours is rare (approximately 4% of autopsy studies) [22,23]. In the present cohort, high-grade PanINs were only present in patients with PDAC. A previous study using a *Kras*^G12D^ transgenic mouse model demonstrated that tuft cells decreased from low- to high-grade PanIN and that prostaglandins secreted from tuft cells were important for inhibiting the acceleration of high-grade PanIN and PDAC [16]. The results of the present study, showing a significant decrease in tuft cells in human high-grade PanIN compared to low-grade PanIN, are in line with those of a previous study on a mouse model [16,24]. The mechanism underlying the decrease in tuft cells in high-grade PanIN remains unclear. However, one hypothesis has been proposed: proliferating high-grade tumour cells may inhibit the differentiation of progenitors into tuft cells or crowd out tuft cells during rapid expansion [25]. Additional studies are needed to elucidate the molecular mechanism underlying the decline of tuft cells in high-grade PanIN because elucidation of this phenomenon might lead to an understanding of the mechanism of progression of high-grade PanIN and PDAC.

This study had some limitations. First, this study included relatively few patients with pancreatic tumours, especially for high-grade PanIN lesions; this could have led to a selection bias. Moreover, the number of patients with PDAC in this cohort was small because over half of the patients with PDAC underwent neoadjuvant chemotherapy and/or radiation therapy at our institute and were excluded from this study. In addition, high-grade PanINs are typically present in the pancreas in PDAC; therefore, we included a relatively small number of high-grade PanIN lesions. Validation studies with larger numbers of patients with PDAC are needed to overcome statistical bias. Moreover, the present cohort included patients with different types of pancreatic tumours; therefore, the type of pancreatic tumour may have influenced local inflammation, tissue repair, and emergence of tuft cells in the pancreas. Second, although the presence of tuft cells in human ADM and PanINs was demonstrated in this study, the origin of tuft cells and secretion of physiologically active substances from tuft cells in ADM and PanINs, which may have influence on immune cells and/or stromal cells, as well as tumour microenvironment, were not evaluated. Further studies are required to clarify these issues in human pancreatic tuft cells. Third, the histopathological criteria for ADM and PanIN and the counts of tuft cells using immunohistochemical staining for POU2F3 might be biased. However, two researchers independently evaluated the histopathological and immunohistochemical features; when there were discrepancies, a final decision was established by reassessment using a multi-headed microscope. Finally, the present study used POU2F3 as a human tuft cell marker, and the immunostainings for other markers, such as Double cortin-like kinase 1 (DCLK1), were not performed. Although DCLK1 has been widely used as a marker for mouse tuft cells [25], it is not suitable for identifying human tuft cells [10,26]. Moreover, POU2F3 is a master regulator of tuft cell identity [10,11], therefore, we used it as a human tuft cell marker.

## 5. Conclusions

This study detected tuft cells in ADM lesions of the human pancreas, which may contribute to inflammation resolution and epithelial regeneration following injury. Moreover, the significantly higher frequency of tuft cells in low-grade PanIN than in high-grade PanIN suggests a potential role in tumorigenesis and suppression of tumour progression in the human pancreas. Further studies are warranted to elucidate the origin of tuft cells and their secretion of physiologically active substances in ADM and PanIN, which may provide new insights into the mechanisms of tumorigenesis in human PDAC.

## Figures and Tables

**Table 1:** Clinicopathological features of the present cohort.

		N = 29 (%)
Age, years, median (range)		74 (51–84)
Sex		
	Men	13 (44.8)
	Women	16 (55.2)
Tumour location		
	Head	11 (37.9)
	Body and tail	18 (62.1)
Tumour histopathology		
	PDAC	7 (24.1)
	IPMN with low-grade dysplasia	10 (34.5)
	IPMN with high-grade dysplasia or associated invasive carcinoma	10 (34.5)
	SPN	1 (3.4)
	NET and IPMN with low-grade dysplasia	1 (3.4)

IPMN, intraductal papillary mucinous neoplasm; NET, neuroendocrine tumour; PDAC, pancreatic ductal adenocarcinoma; SPN, solid pseudopapillary neoplasm.

**Table 2:** Summary of the immunohistochemical results.

	POU2F3-Positive Tuft Cells per PanIN Area (Median)	Percentage of POU2F3-Positive Tuft Cells per Total Cells (Median)
Low-grade PanIN	2 cells (range, 0–20)	1.1% (range, 0–2.5%)
High-grade PanIN	0 cell (range, 0–4)	0% (range, 0–1.1%)
	***p* = 0.0050**	***p* = 0.0044**

PanIN, Pancreatic intraepithelial neoplasia; POU2F3, POU domain class 2 transcription factor 3.

## Data Availability

Due to the nature of this research, participants in this study could not be contacted about whether the findings could be shared publicity. Thus, supporting data are not available. The datasets generated and analysed during the current study are not publicly available due to the nature of the research but are available from the corresponding author on reasonable request.

## Abbreviations

The following abbreviations are used in this manuscript:

ADM	Acinar-to-ductal metaplasia
DCLK1	Double cortin-like kinase 1
IPMN	Intraductal papillary mucinous neoplasm
PanIN	Pancreatic intraepithelial neoplasia
PDAC	Pancreatic ductal adenocarcinoma
POU2F3	POU domain class 2 transcription factor 3
